# The Impact of Chronic Diseases on the Quality of Life of Primary Care Patients in Cambodia, Myanmar and Vietnam

**Published:** 2018-09

**Authors:** Supa PENGPID, Karl PELTZER

**Affiliations:** 1. ASEAN Institute for Health Development, Mahidol University, Salaya, Phutthamonthon, Nakhonpathom, Thailand; 2. Dept. of Research Innovation and Development, University of Limpopo, Sovenga, South Africa; 3. Dept. for Management of Science and Technology Development, Ton Duc Thang University, Ho Chi Minh City, Vietnam; 4. Faculty of Pharmacy, Ton Duc Thang University, Ho Chi Minh City, Vietnam

**Keywords:** Chronic disease patients, Quality of life, Primary health care, ASEAN

## Abstract

**Background:**

Quality of life is a key measure in estimating the burden of disease, especially of chronic diseases. This study investigated the impact of a variety of chronic diseases on quality of life (QoL) in primary health care patients in three Southeast Asian countries (Cambodia, Myanmar, and Vietnam).

**Methods:**

This cross-sectional survey was conducted on 4803 adult chronic disease patients (mean age 49.3 yr; SD=16.5) recruited systematically from primary health care centers in rural and urban areas in Cambodia, Myanmar and Vietnam in 2015.

**Results:**

In ANCOVA analysis, adjusted for age, sex, marital status, geo locality, education, income and country, the poorest summative QoL was found among patients with cancer (49.8 mean score), followed by Parkinson’s disease (50.7), mental disorder (53.2), epilepsy (53.3), asthma (54.3), kidney disease (54.3), chronic obstructive pulmonary disease (COPD) (54.5) and cardiovascular diseases (CVD) (55.1). Patients having three or more chronic conditions had a significantly lower summative QoL than patients with two chronic conditions (56.4) and one chronic condition (58.0). In multivariable linear regression analysis, younger age, being married or cohabitating, better education, living in an urban area, having only one chronic condition, not experiencing chronic disease stigma and good medication adherence was associated with better QoL in two or three of the study countries.

**Conclusion:**

Major chronic diseases were found to have poor QoL. The determined QoL of chronic disease patients in this study provides information to improve the management of chronic diseases.

## Introduction

Chronic diseases (1), including cardiovascular diseases (CVD) (2–5), hypertension (3, 6), diabetes (4, 5, 7), cancer (4, 5), chronic obstructive pulmonary disease (COPD) (5, 8), asthma (2), kidney disease (9) and musculoskeletal diseases (10), have been commonly reported among the aging population in Southeast Asian countries, including Cambodia, Myanmar and Vietnam. In low and middle-income countries such as in Cambodia, Myanmar and Vietnam primary health care are (11), the most available healthcare service to cater for the long-term needs of chronic disease patients. There is a lack of studies, in particular in Southeast Asia, on the effects of chronic diseases on the quality of life (QoL) in primary care patients. Since primary health care services have a key role in chronic care management, the identification of the levels of QoL of chronic disease patients can potentially provide useful information in improving health services for these patients (12).

One study among hypertensive patients (50 yr and older) in Vietnam found that the QoL was moderate in the domains of physical (mean=54.7), social relationships (64.1) and environment (59.5), and low for psychological domain (mean = 49.4) (13). Among Chinese primary care patients, depression and osteoarthritis of the knee had the most negative impact on QoL compared to many other chronic diseases (14). Similarly, among primary care patients in Turkey, mental disorders and diabetes-hypertension comorbidity had the most negative effect on the QoL (12). Among seven chronic conditions, arthritis, COPD and CVD had the lowest QoL scores in a multi-country study (15), while in a Canadian study “arthritis, heart disease, high blood pressure, cataracts, and diabetes had the most severe impact on QoL, urinary incontinence, Alzheimer/other dementia, effects of stroke, cancers, thyroid condition, and back problems had a moderate impact on QoL, and food allergy, allergy other than food, asthma, migraine headaches, and other remaining chronic diseases had a relatively mild effect on QoL” (16).

Reviews and specific studies found that lower QoL was associated multimorbidity or comorbidity (12, 13, 17, 18), increasing age (13, 18–20), being female (13, 19, 20), not married or cohabiting (13, 19, 20), lack of higher education (13, 18–20), lower income or socioeconomic status (12, 20, 21), rural residence (19); smoking (18), not currently drinking alcohol (22) and lack of adherence to treatment (13).

This study investigated the impact of a variety of chronic diseases on QoL in primary health care patients in three Southeast Asian countries (Cambodia, Myanmar, and Vietnam).

## Materials and Methods

### Sample and procedure

In each Southeast Asian country (Cambodia, Myanmar, and Vietnam), a cross-sectional survey was conducted in rural and urban primary health care facilities with out-patients with chronic diseases in 2015. The sample size included at least 800 people from rural health facilities and 800 individuals from urban health facilities in each country (23).

Every eligible patient (18 yr and older treated for a chronic disease in the past 12 months) was selected from the health facility, using a convenient sampling procedure (consecutively selecting every out-patient visiting the health facility) (23). Trained research assistants conducted interviews with the patients using structured questionnaires after informed consent was obtained (23). We recruited all of the patients who accessed the services of the selected health facilities for their treatment with some inclusion criteria, including adult patients with a minimum age of 18 yr treated in the past 12 months for any of the 21 chronic conditions, such as asthma, chronic obstructive pulmonary disease (COPD), diabetes mellitus, hypertension, dyslipidemia, coronary artery disease, cardiac failure, cardiac arrhythmias, stroke, arthritis, cancer, gout and other musculoskeletal conditions, such as chronic backache, Parkinson’s disease, liver disease, kidney disease, thyroid disease, stomach and intestinal diseases, epilepsy and mental disorders (23, 24). The questionnaire had been translated and back-translated by certified translators into the study languages, Myanmar, Khmer, and Vietnamese (23).

In Cambodia, the National Ethics Committee for Health Research of the Ministry of Health (Reference no: 0225NECHR); in Myanmar, the Research and Ethical Committee of University of Medicine 1, Yangon; in Vietnam, the Committee of Research Ethics of Hanoi School of Public Health; and in Thailand, the Committee of Research Ethics (Social Sciences) of Mahidol University (COA. No.: 2014/193.0807) approved the study protocol (23).

### Measures

Qol was assessed with the World Health Organization Quality of Life (WHOQol)-8 consisting of eight items derived from the WHOQOL-Bref (25). The 8-item index “consists of two items from each domain of the original WHOQOL-BREF (physical, psychological, environmental, and social) (26) and showed acceptable cross-cultural performance and a satisfactory discriminant validity (26). Results from the 2-items sub-scales and the 8-items were summed to get sub-scale and overall WHOQoL scores which was then transformed to a 0–100 scale. The Cronbach alpha for the WHOQol-8 was 0.89 in this sample. Sociodemographic information included age, sex, country, formal education, marital status, income, and residency status.

Anticipated stigma was assessed with the 12-item “Chronic Illness Anticipated Stigma Scale” (CIASS) (27). The CIASS is calculated by adding up all items, and in this study, the median was calculated, with 21 or more indicating anticipated chronic disease stigma. The Cronbach alpha for the CIASS in this study was 0.91.

Problem drinking was assessed with the “Alcohol Use Disorder Identification Test” (AUDIT)-C, using a cut-off score of four for problem drinking (28). The Cronbach alpha for the AUDIT-C was 0.81 in this sample.

Current smoking was assessed with the following question: 1) “Do you currently smoke any tobacco products, such as cigarettes, cigars or pipes?”. Response options were “yes” or “no” (29).

The 8-item “Morisky Medication Adherence Scale” (MMAS) was used to assess medication adherence for specific chronic illnesses (30), e.g., “Do you feel hassled by sticking to your treatment plan?” Total scores from this scale range from 0 to 8, with scores of 6–8 as medium or high adherence (good adherence) (30).

### Data analyses

Data analysis was performed using STATA software version 13.0 (Stata Corporation, College Station, Texas, USA). The descriptive characteristics of the study population were calculated as a percentage. Analysis of covariance (ANCOVA) was utilized to assess the associations between five measures of QoL (the four QoL domains: Psychological, Physical, Social and Environment, and the summative QoL) and various chronic diseases and adjustments were made for age, gender, marital status, education, income, locality, comorbidity and country. Multi-variable linear regression was used for the assessment of the impact of explanatory variables (socio-demographic factors, number of chronic diseases, chronic disease stigma, problem drinking, smoking and medication adherence) for summative QoL for each country separately. The *P*-value of less than 5% is used to indicate statistical significance.

## Results

### Sample characteristics

The total sample included 4803 adults (1602 in Cambodia, 1600 in Myanmar and 1601 in Vietnam), the mean age was 49.3 yr (SD=16.5), the range was 18–101 yr. In the three study countries, all study participants approached agreed to participate (response rate=100%). In all three countries, there were more female than male participants. The proportion of 61 yr and older participants was highest in Myanmar (39.2%) compared to below 20% in Cambodia and Vietnam. Participants with Grade 0–5 education was the highest in Cambodia (64.4%) and with Grade 12 or more education was the highest in Vietnam (46.6%). In Myanmar and Vietnam, about two-thirds of the participants were suffering from one chronic condition, while in Cambodia 60.4% had two or more chronic conditions. Among the three countries, problem drinking was the highest in Vietnam (19.8%) and current smoking was the highest in Myanmar (20.4%). Good adherence to chronic disease medication was the highest in Cambodia (68.4%) and the lowest in Vietnam (29.5%) (Table 1).

**Table 1:** Descriptive characteristics of study population in Cambodia, Myanmar, and Vietnam

***Characteristics Variable***	***Cambodia (n=1602) N (%)***	***Myanmar (n=1600) N (%)***	***Vietnam (n=1601) N (%)***
Sex			
Female	1241 (77.7)	1118 (69.9)	955 (61.1)
Male	356 (22.3)	482 (30.1)	608 (38.9)
Age (yr)			
18–45	754 (47.1)	405 (25.3)	758 (52.3)
46–60	544 (34.0)	568 (35.5)	405 (28.0)
61–101	304 (19.0)	627 (39.2)	285 (19.7)
Marital status			
Single/divorced/widowed	345 (22.0)	466 (29.1)	438 (28.0)
Married/cohabiting	1220 (78.0)	1134 (70.9)	1124 (72.0)
Education			
Grade 0–5	1030 (64.4)	503 (31.4)	117 (7.3)
Grade 6–11	445 (27.8)	900 (56.3)	735 (46.0)
Grade 12 or more	125 (7.8)	197 (12.3)	745 (46.6)
Income			
Low	993 (62.9)	1168 (75.3)	1075 (67.1)
High	586 (37.1)	384 (24.7)	526 (32.9)
Geolocality			
Rural	1017 (63.9)	800 (50.0)	840 (52.5)
Urban	575 (36.1)	800 (50.0)	760 (47.5)
Chronic conditions			
One	633 (39.6)	1031 (64.4)	941 (59.3)
Two	520 (32.6)	431 (26.9)	407 (25.7)
Three or more	444 (27.8)	138 (8.6)	238 (15.0)
Chronic disease stigma			
No	1038 (64.8)	811 (50.7)	494 (30.9)
Yes	564 (35.2)	789 (49.3)	1107 (69.1)
Problem drinking			
No	1518 (94.8)	1556 (97.3)	1284 (80.2)
Yes	84 (5.2)	44 (2.8)	317 (19.8)
Current smoking			
No	1434 (89.7)	1274 (79.6)	1287 (83.1)
Yes	165 (10.3)	326 (20.4)	261 (16.8)
Adherence			
Poor	411 (31.6)	749 (46.8)	1013 (70.5)
Good	889 (68.4)	851 (53.2)	424 (29.5)

### QoL by specific chronic disease

In ANCOVA analysis, adjusted for age, sex, marital status, geolocality, education, income, comorbidity and country, the poorest summative QoL was found among patients with cancer (49.8 mean score), followed by Parkinson’s disease (50.7), mental disorder (53.2), epilepsy (53.3), asthma (54.3), kidney disease (54.3), COPD (54.5) and CVD (55.1).

The highest summative QoL score was found among patients having dyslipidemia (63.2), followed by digestive diseases (57.7), liver disease (57.5), hypertension (57.4) and diabetes mellitus (57.1). Patients having three or more chronic conditions had a significantly lower summative QoL than patients with two chronic conditions (56.4) and one chronic condition (58.0).

The overall summative score of the QoL was 57.1, while the domain QoL-Social had the highest score 61.2, followed by QoL-Psychological 57.1, and the lowest domain score was QoL-Environment 53.9, followed by QoL-Physical 55.8. QoL-Psychological had the lowest scores for cancer (49.0), Parkinson’s disease (50.7), thyroid disorder (53.0), epilepsy (53.7) and mental disorder (53.7). QoL-Physical was the lowest for cancer (46.3), epilepsy (50.3), Parkinson’s disease (50.9), mental disorder (51.7) and CVD (51.8). QoL-Social was the lowest for cancer (53.6), epilepsy (55.7) and Parkinson’s disease (57.3), while QoL-Environment was the lowest for Parkinson’s disease (44.7), mental disorder (48.0), kidney disease (49.8) and COPD (49.9). With multi-comorbidity, the scores for the four QoL domain scores decreased, while they significantly decreased for QoL-Physical, QoL-Environment and the summative QoL (Table 2).

**Table 2:** Adjusted Quality of Life (QoL) according to chronic disease

***Chronic condition***	***Sample***	***QoL-Psychological***	***QoL-Physical***	***QoL-Social***	***QoL-Environment***	***QoL-total***
	***N***	***Mean (95% CI)***	***Mean (95% CI)***	***Mean (95% CI)***	***Mean (95% CI)***	***Mean (95% CI)***
**All**	4803	57.1 (56.7, 57.6)	55.8 (55.3, 56.3)	61.2 (60.9, 61.7)	53.9 (53.5, 54.3)	57.1 (56.7, 57.4)
Digestive diseases	1935	58.5 (57.8, 59.2)^*^	57.6 (56.8, 58.3)^*^	62.9 (62.2, 63.6)^*^	51.5 (50.8, 52.2)^*^	57.7 (57.1, 58.2)
Hypertension	1402	58.0 (57.2, 58.9)	55.5 (54.6, 56.4)	61.4 (60.6, 62.3)	54.6 (53.8, 55.4)	57.4 (56.8, 58.1)
Arthritis	909	57.1 (56.1, 58.1)	54.3 (53.2, 55.5)	60.5 (59.5, 61.5)	52.4 (51.4, 53.4)	56.1 (55.3, 56.9)
Cardiovascular diseases^1^	804	55.4 (54.3, 56.4)^*^	51.8 (50.6, 52.9)^*^	59.8 (58.8, 60.9)^*^	53.3 (52.2, 54.3)	55.1 (54.2, 55.9)^*^
Musculoskeletal diseases	748	55.4 (54.2, 56.5)^*^	55.6 (59.5, 61.7)	60.6 (59.5, 61.7)	53.3 (52.2, 54.4)	56.2 (55.3, 57.1)
Diabetes mellitus	509	57.8 (56.5, 59.2)	54.5 (53.0, 55.9)	61.1 (59.8, 62.4)	54.9 (53.5, 56.2)	57.1 (56.0, 58.2)
Migraine or frequent headaches	350	56.6 (54.9, 58.2)	54.7 (52.9, 56.5)	61.6 (60.0, 63.2)	52.5 (50.9, 54.1)	56.5 (55.2, 57.8)
Chronic obstructive pulmonary disease (COPD)	308	55.5 (53.8, 57.2)	52.7 (50.8, 54.5)^*^	59.9 (58.2, 61.5)	49.9 (48.2, 51.6)	54.5 (53.1, 55.9)^*^
Kidney disease	240	55.4 (53.4, 57.3)	52.8 (50.7, 55.1)	59.2 (57.3, 61.2)	49.8 (47.9, 51.7)^*^	54.3 (52.7, 55.9)^*^
Liver disease	234	56.8 (54.9, 58.8)	56.6 (54.4, 58.8)	61.3 (59.4, 63.3)	54.6 (52.7, 56.6)	57.5 (55.9, 59.1)
Asthma	219	54.2 (52.2, 56.2)	52.2 (49.9, 54.4)^*^	58.6 (56.6, 60.6)	53.2 (51.1, 55.2)	54.5 (52.8, 56.1)^*^
Dyslipidemia	210	64.2 (62.1, 66.3)^*^	65.2 (62.8, 67.5)^*^	66.2 (64.2, 68.3)^*^	57.0 (54.9, 59.0)	63.2 (61.5, 64.9)^*^
Mental disorder	119	53.7 (50.9, 56.4)	51.7 (48.6, 54.7)	58.9 (56.2, 61.6)	48.0 (45.3, 50.7)^*^	53.2 (50.9, 55.4)^*^
Thyroid disease	75	53.0 (49.5, 56.5)	54.8 (50.9, 58.8)	60.3 (56.8, 63.9)	55.6 (52.0, 59.0)	56.2 (53.2, 59.1)
Cancer	72	49.0 (45.4, 52.6)^*^	46.3 (42.3, 50.2)	53.6 (50.1, 57.1)	50.1 (46.6, 53.7)	49.8 (46.9, 52.7)^*^
Parkinson’s disease	69	50.7 (47.0, 54.4)^*^	50.9 (46.8, 58.0)	57.3 (53.7, 60.9)	44.7 (41.0, 48.3)^*^	50.7 (47.7, 53.7)^*^
Epilepsy	16	53.7 (46.5, 60.8)	50.3 (42.4, 58.2)	55.7 (48.7, 62.8)	53.7 (46.5, 60.8)	53.3 (47.6, 59.1)
**Number of chronic conditions**						
One chronic condition	2605	57.4 (56.9, 58.0)	57.2 (56.6, 57.9)	61.8 (61.3, 62.4)	55.2 (54.6, 55.8)	58.0 (57.5, 58.4)
Two chronic conditions	1358	57.0 (56.2, 57.8)	54.5 (53.6, 55.4)	60.6 (59.9, 61.4)	53.1 (52.3, 53.9)	56.4 (55.7, 57.0)
Three or more chronic conditions	840	56.3 (55.3, 57.3)	53.2 (52.1, 54.4)^*^	60.7 (59.7, 61.7)	50.9 (49.9, 52.0)^*^	55.3 (54.5, 56.2)^*^

1 Cardiac failure, Coronary artery disease, Cardiac arrhythmias, Stroke; Adjusted for age, sex, marital status, geolocality, education, income, comorbidity, and country;

* *P*<0.001

### Associations with QoL

In multi-variable linear regression analysis, a significant association was observed between being male, younger age (18–45 yr), being married or cohabiting, Grade 12 or more education, higher income, having fewer chronic conditions, not experiencing chronic disease stigma and better QoL in Cambodia. Better education, living in an urban area, not using tobacco and good adherence to chronic disease medication was associated with better QoL in Myanmar. Younger age, having Grade 12 or more education, living in an urban area, having only one chronic condition, not experiencing chronic disease stigma and good medication adherence was associated with better QoL in Vietnam (Table 3).

**Table 3:** Factors associated with summative quality of life

***Variable***	***Cambodia***		***Myanmar***		***Vietnam***	
	***B (95% confidence interval)***	***P***	***B (95% confidence interval)***	***P***	***B (95% confidence interval)***	***P***
Sex						
Female	Reference		Reference		Reference	
Male	1.79 (0.18, 3.39)	0.029	−1.04 (−2.60, 0.52)	0.118	1.59 (−0.89, 4.06)	0.220
Age (yr)						
18–45	Reference		Reference		Reference	
46–60	−1.64 (−3.23, −0.05)	0.011	0.90 (−0.63, 2.43)	0.249	−0.99 (−2.57, 0.60)	0.471
61–101	−3.51 (−6.61, −1.40)	<0.001	0.97 (−0.53, 2.47)	0.206	−2.94 (−4.74, −1.14)	<0.001
Marital status						
Married/cohabiting	Reference		Reference		Reference	
Single/divorced/widowed	−2.37 (−4.08, −0.66)	0.008	−1.24 (−2.81, 0.33)	0.187	−0.07 (−2.23, 2.09)	0.971
Education						
Grade 0–5	Reference		Reference		Reference	
Grade 6–11	2.88 (1.43, 4.33)	<0.001	0.82 (−0.45, 2.09)	0.605	1.55 (−1.20, 4.30)	0.259
Grade 12 or more	7.34 (4.88, 9.81)	<0.001	6.16 (4.20, 8.14)	<0.001	4.67 (1.81, 7.54)	<0.001
Income						
Low	Reference		Reference		Reference	
High	1.93 (0.59, 3.26)	0.003	0.64 (−0.88, 2.15)	0.355	0.64 (−1.53, 2.81)	0.754
Geolocality						
Rural	Reference		Reference		Reference	
Urban	1.61 (−0.18, 3.40)	0.098	5.70 (3.87, 7.73)	<0.001	3.05 (0.99, 5.10)	0.007
Chronic conditions						
One	Reference		Reference		Reference	
Two	−1.85 (−3.53, −0.17)	0.027	−0.009 (−1.70, 1.68)	0.911	−2.97 (−4.43, −1.52)	<0.001
Three or more	−2.73 (−4.62, −0.85)	0.009	2.12 (−0.41, 4.64)	0.090	−3.33 (−5.11, −1.55)	<0.001
Chronic disease stigma						
No	Reference		Reference		Reference	
Yes	−7.54 (−9.35, −5.73)	<0.001	0.52 (−0.91, 1.95)	0.450	−6.23 (−8.31, −4.16)	<0.001
Problem drinking						
No	Reference		Reference		Reference	
Yes	1.33 (−1.56–4.20)	0.195	−1.65 (−6.36, 3.06)	0.295	−1.79 (−1.01, 4.59)	0.151
Tobacco use						
No	Reference		Reference		Reference	
Yes	−0.03 (−2.39−2.33)	0.081	−2.17 (−4.00, −0.35)	0.040	0.68 (−2.37, 3.73)	0.695
Adherence						
Poor	Reference		Reference		Reference	
Good	1.20 (1.51, −1.65)	0.835	4.61 (3.41, 5.81)	<0.001	3.19 (1.66, 4.71)	<0.001

## Discussion

To our knowledge, this is one of the first studies investigating the impact of chronic diseases on QoL in primary care patients in Southeast Asia. Among chronic disease patients with a variety of chronic illnesses across three Southeast Asian countries, that the poorest summative QoL was found among patients with cancer (mean=49.8), followed by Parkinson’s disease (50.7), mental disorder (53.2), epilepsy (53.3), asthma (54.3), kidney disease (54.3), COPD (54.5), CVD (55.1) and arthritis (56.1). Mental disorders, COPD, arthritis, and CVD had the most negative impact on QoL compared to many other chronic diseases, probably due to their more symptomatic and disabling nature (12, 14–16). On the other hand, patients reporting more asymptomatic or less disabling conditions, such as hypertension and dyslipidemia, had better QoL scores, as also found in previous studies (e.g., 15). Further, different chronic diseases affected specific domains of QoL differently, as also found previously (14, 31). Having a mental disorder was not only impacting negatively on psychological QoL but also physical QoL, which may have the implication of managing mental and physical problems concurrently (14).

In comparison to a study among hypertensive patients (50 yr and older) in Vietnam, this study found among hypertensive patients similar values for the QoL domains Physical, Social and Environment but higher values for Psychological (58.0, as opposed to 49.4) in the Vietnam study (13). Further, in agreement with previous studies (12, 13, 17, 18), with multimorbidity the scores for QoL scores decreased. However, the contribution of multimorbidity on QoL-Psychological and QoL-Social was not significant, as also noted in a previous review in terms of a less clear association (17).

In agreement with previous studies (13, 18–20), we found that sociodemographic variables (younger age, being married or cohabitating, better education and living in an urban area) were associated with better QoL scores. Older age may be associated with a decline in physical and mental capabilities (32), which may explain the lower QoL in older patients in Cambodia and Vietnam. However, this difference was not observed among our study participants in Myanmar. Higher education and living in an urban area may be associated with better knowledge and access to health matters and services, consequently leading to better health and QoL (32). Being married or cohabiting has a vital role in social support, as opposed to living alone, and may facilitate QoL (32). Good medication adherence was also associated with better QoL in two of three of the study countries (13). Not experiencing chronic disease stigma was in two study countries (Cambodia and Vietnam) highly associated with better QoL. Further, in agreement with some previous studies (13, 19, 20), compared to men, women had a lower QoL in Cambodia, while there was no gender difference in Myanmar and Vietnam. Unlike some previous studies (18, 22), this study found a lack of association between smoking, not currently drinking alcohol and QoL.

This study had several limitations. The study was cross-sectional; therefore, causal conclusions cannot be drawn. The investigation was carried out with chronic disease patients from conveniently selected primary health facilities in the study countries, who tended compared to specialist care have probably milder or more stable conditions. Therefore the inclusion of other or specialist health facilities could have produced different results. Another one was that all the other information collected in the study was based on self-reporting. Some of the included chronic diseases such as epilepsy in the study had small subgroup sample sizes, which limited the detection of associations.

## Conclusion

Major chronic diseases were found to have poor QoL, especially cancer, Parkinson’s disease, mental disorder, epilepsy, asthma, kidney disease, COPD, CVD, and arthritis. The determined QoL of chronic disease patients and the identified factors of influencing QoL such as sociodemographics, comorbidity, chronic disease stigma and poor medication adherence in this study provide information to improve the management of chronic diseases in this Southeast Asian setting.

## Ethical considerations

Ethical issues (Including plagiarism, informed consent, misconduct, data fabrication and/or falsification, double publication and/or submission, redundancy, etc.) have been completely observed by the authors.
